# Supplementary material for: Serum Amyloid A is not obligatory for high-fat, high-sucrose, cholesterol-fed diet-induced obesity and its metabolic and inflammatory complications
Source: PLoS One. 2022 Apr 18;17(4):e0266688. doi: 10.1371/journal.pone.0266688 (PMC9015120; doi:10.1371/journal.pone.0266688)
Supplement: S1 Fig — A) Genotyping to identify SAA TKO mice are done by a multi-step process. The first step is genotyping for SAA1/2 KO utilizing a 3 primer PCR reaction as described earlier (de Beer et al., J Lipid Res. 2010. 51:3117–3125). The genotype to determine SAA 3 deficiency is performed by two sets of PCRs, using forward primer for the PCRs designed to detect WT sequence or SAA3 KO (CRISPR-Cas9) sequences respectively. The primers for the two PCRs are shown on the right panel. The generation of TKO mice from SAA1.1/SAA2.1-deficient mice is described earlier [35]. B) Expression of SAA3 mRNA in the adipose tissues of male (left panel) and female (right panel) WT and TKO mice fed either chow or HFHSC diet for 16 weeks. C) SAA4 mRNA expression in the adipose tissues of male (left panel) and female (right panel) WT and TKO mice fed either chow or HFHSC diet for 16 weeks. D) Expression of SAA3 mRNA in the livers of male (left panel) and female (right panel) WT and TKO mice fed either chow or HFHSC diet for 16 weeks. Data are mean ±SEM; ** = P ≤ 0.01 and *** = P ≤ 0.001. (PPTX) [file pone.0266688.s001.pptx]

## Slide 1
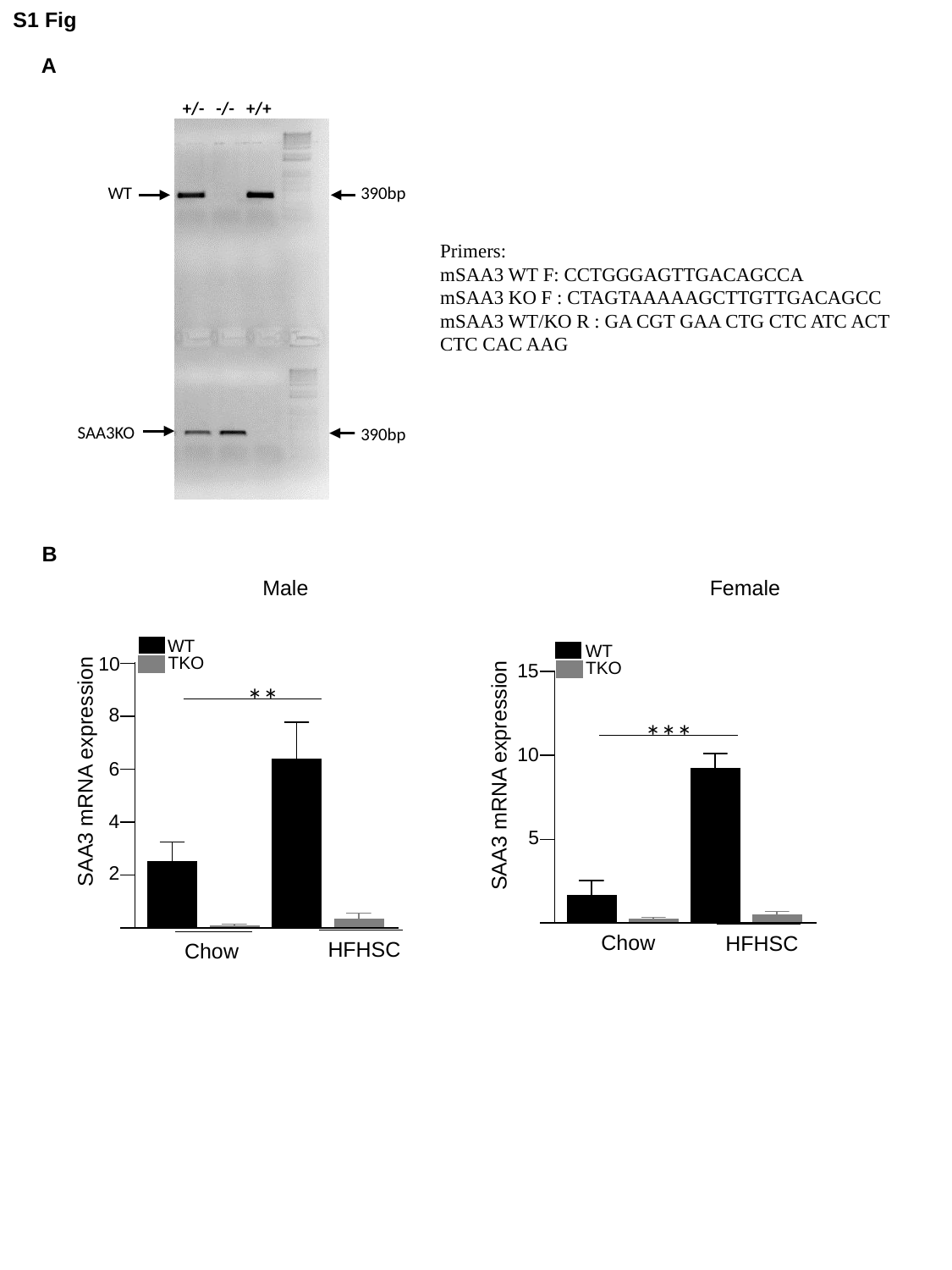

S1 Fig
A
+/- -/- +/+
WT
390bp
SAA3KO
390bp
Primers:
mSAA3 WT F: CCTGGGAGTTGACAGCCA
mSAA3 KO F : CTAGTAAAAAGCTTGTTGACAGCC
mSAA3 WT/KO R : GA CGT GAA CTG CTC ATC ACT CTC CAC AAG
B
Male
Female
WT
TKO
10
**
8
6
SAA3 mRNA expression
4
2
HFHSC
Chow
WT
TKO
15
10
SAA3 mRNA expression
5
Chow
HFHSC
***

## Slide 2
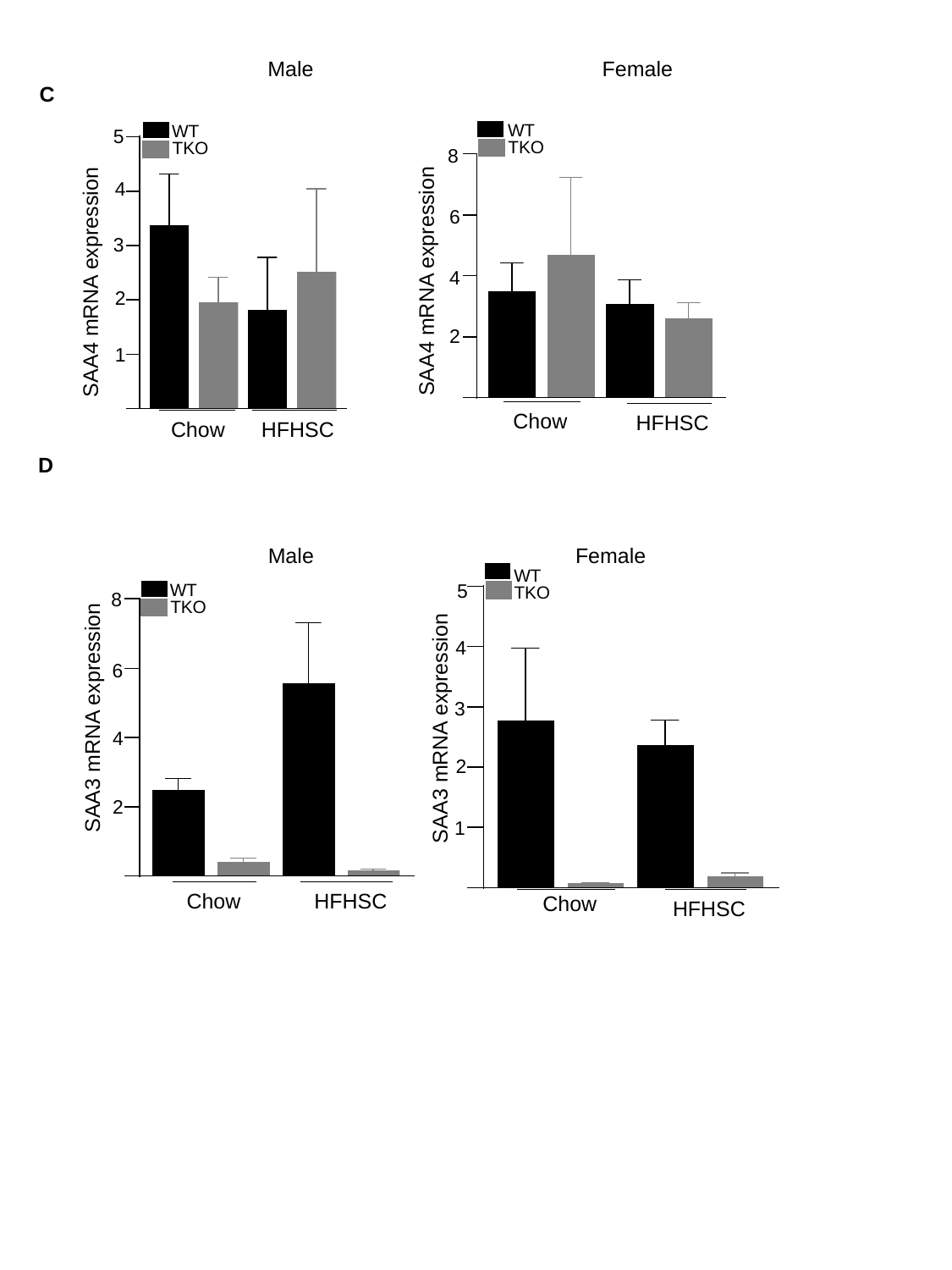

Male
Female
C
WT
TKO
8
6
4
SAA4 mRNA expression
2
Chow
HFHSC
WT
5
TKO
4
3
SAA4 mRNA expression
2
1
Chow
HFHSC
D
WT
5
TKO
4
3
SAA3 mRNA expression
2
1
Chow
HFHSC
WT
8
TKO
6
SAA3 mRNA expression
4
2
Chow
HFHSC
Male
Female
