# Supplementary figures and images for: Serum Amyloid A is not obligatory for high-fat, high-sucrose, cholesterol-fed diet-induced obesity and its metabolic and inflammatory complications
Source: PLoS One. 2022 Apr 18;17(4):e0266688. doi: 10.1371/journal.pone.0266688 (PMC9015120; doi:10.1371/journal.pone.0266688)

## Slide 1
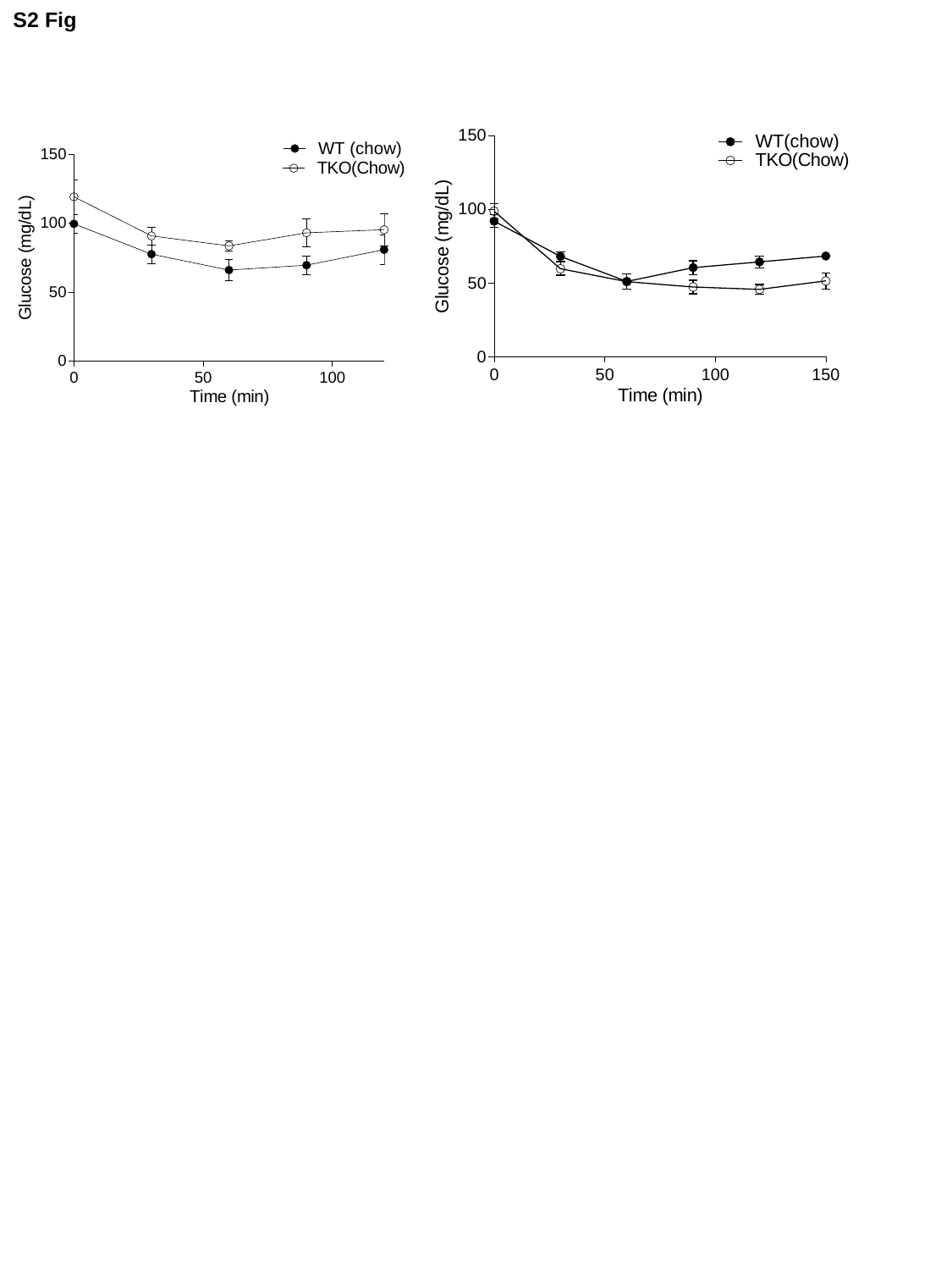

S2 Fig

Supplement: S2 Fig — Insulin tolerance test (ITT) was performed in male (left panel) and female (right panel) WT and TKO mice on chow diet (n = 4–5 mice/group). (PPTX) [file pone.0266688.s002.pptx]
