# Supplementary material for: Serum Amyloid A is not obligatory for high-fat, high-sucrose, cholesterol-fed diet-induced obesity and its metabolic and inflammatory complications
Source: PLoS One. 2022 Apr 18;17(4):e0266688. doi: 10.1371/journal.pone.0266688 (PMC9015120; doi:10.1371/journal.pone.0266688)
Supplement: S3 Fig — Plasma triglycerides A), non-esterified fatty acids B) and total cholesterol C) levels in male (left panel) and female (right panel) WT and TKO mice (n = 4–15) fed either chow or HFHSC diet for 16 weeks. D) Plasma HDL levels in male (left panel) and female (right panel) WT and TKO mice (n = 4–15) fed either chow or HFHSC diet for 16 weeks. Data are mean ±SEM; data that are not significantly different (P>0.05) are indicated with the same letter. (PPTX) [file pone.0266688.s003.pptx]

## Slide 1
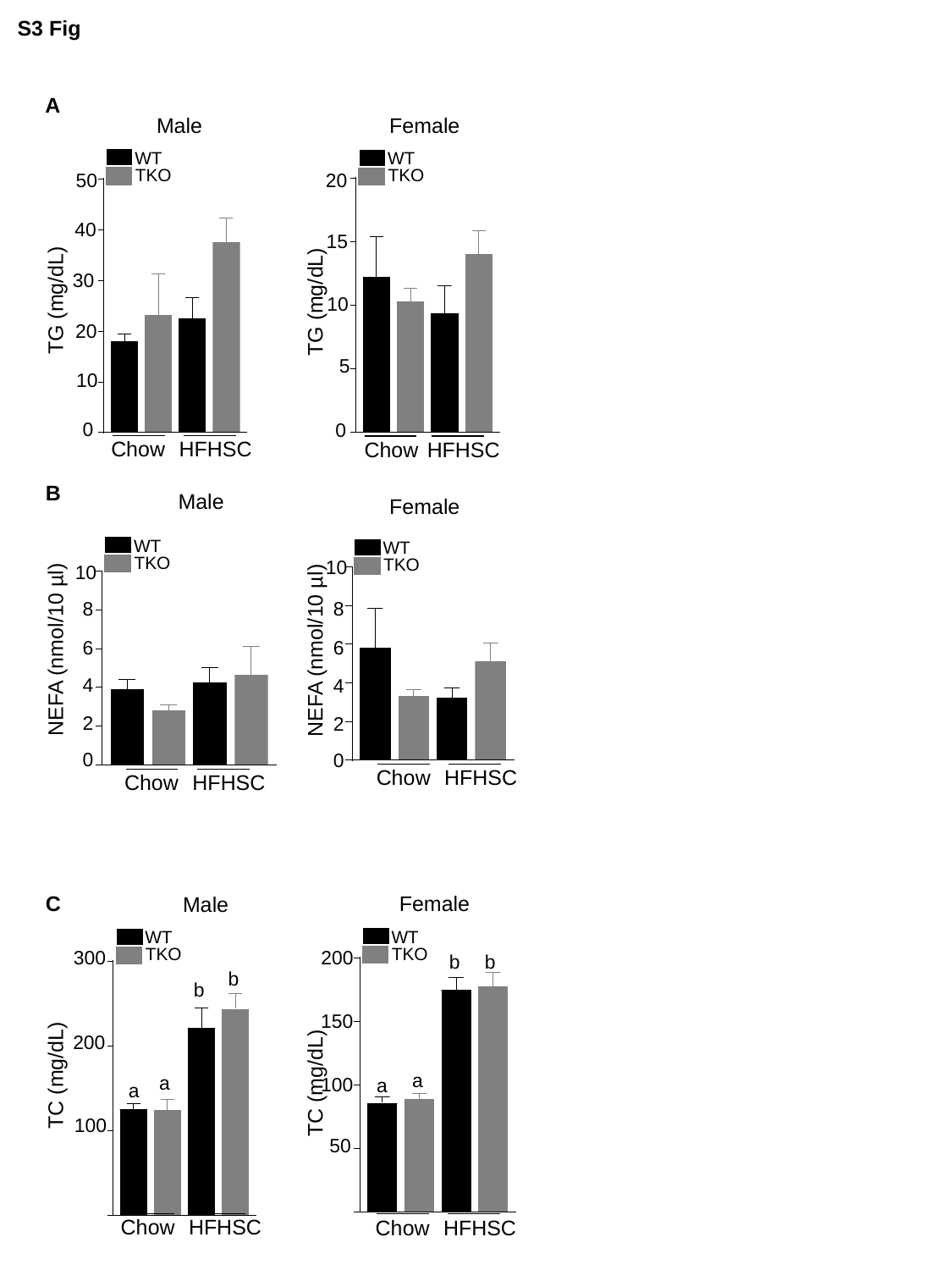

S3 Fig
A
Female
WT
TKO
20
15
TG (mg/dL)
10
5
0
Chow
HFHSC
Male
WT
TKO
50
40
30
TG (mg/dL)
20
10
0
Chow
HFHSC
B
Male
WT
TKO
10
8
6
NEFA (nmol/10 µl)
4
2
0
Chow
HFHSC
Female
WT
TKO
10
8
6
NEFA (nmol/10 µl)
4
2
0
Chow
HFHSC
Female
WT
TKO
200
b
b
150
a
TC (mg/dL)
100
a
50
Chow
HFHSC
C
Male
WT
TKO
300
b
b
200
TC (mg/dL)
a
a
100
Chow
HFHSC

## Slide 2
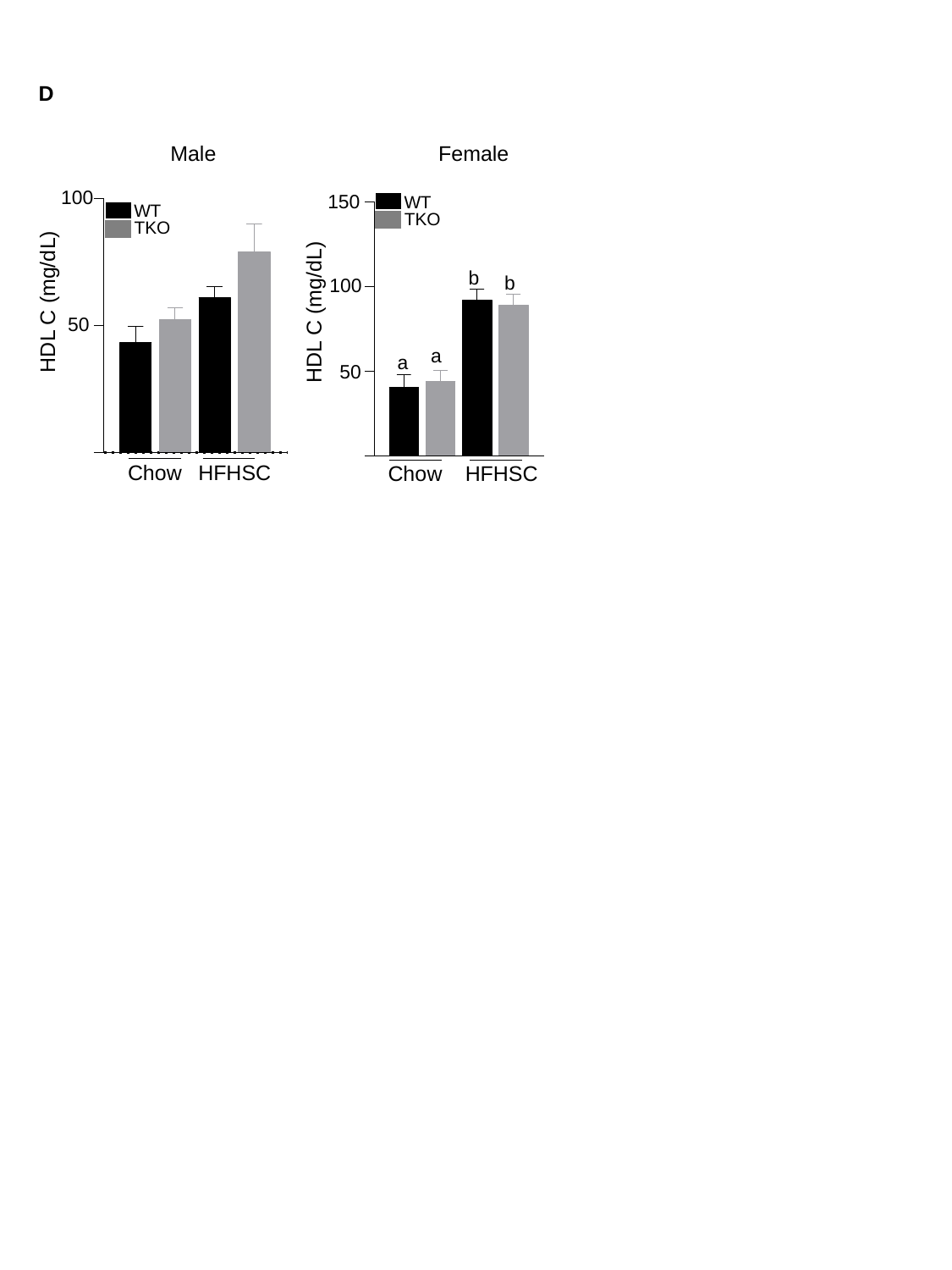

D
Male
Female
100
150
WT
WT
TKO
TKO
b
b
100
HDL C (mg/dL)
HDL C (mg/dL)
50
a
a
50
Chow
HFHSC
Chow
HFHSC
