# Supplementary material for: Serum Amyloid A is not obligatory for high-fat, high-sucrose, cholesterol-fed diet-induced obesity and its metabolic and inflammatory complications
Source: PLoS One. 2022 Apr 18;17(4):e0266688. doi: 10.1371/journal.pone.0266688 (PMC9015120; doi:10.1371/journal.pone.0266688)
Supplement: S4 Fig — Liver triglycerides A) and total cholesterol B) levels in male (left panel) and female (right panel) WT and TKO mice (n = 5–15) fed either chow or HFHSC diet for 16 weeks. Data are mean ±SEM; data that are not significantly different (P>0.05) are indicated with the same letter. (PPTX) [file pone.0266688.s004.pptx]

## Slide 1
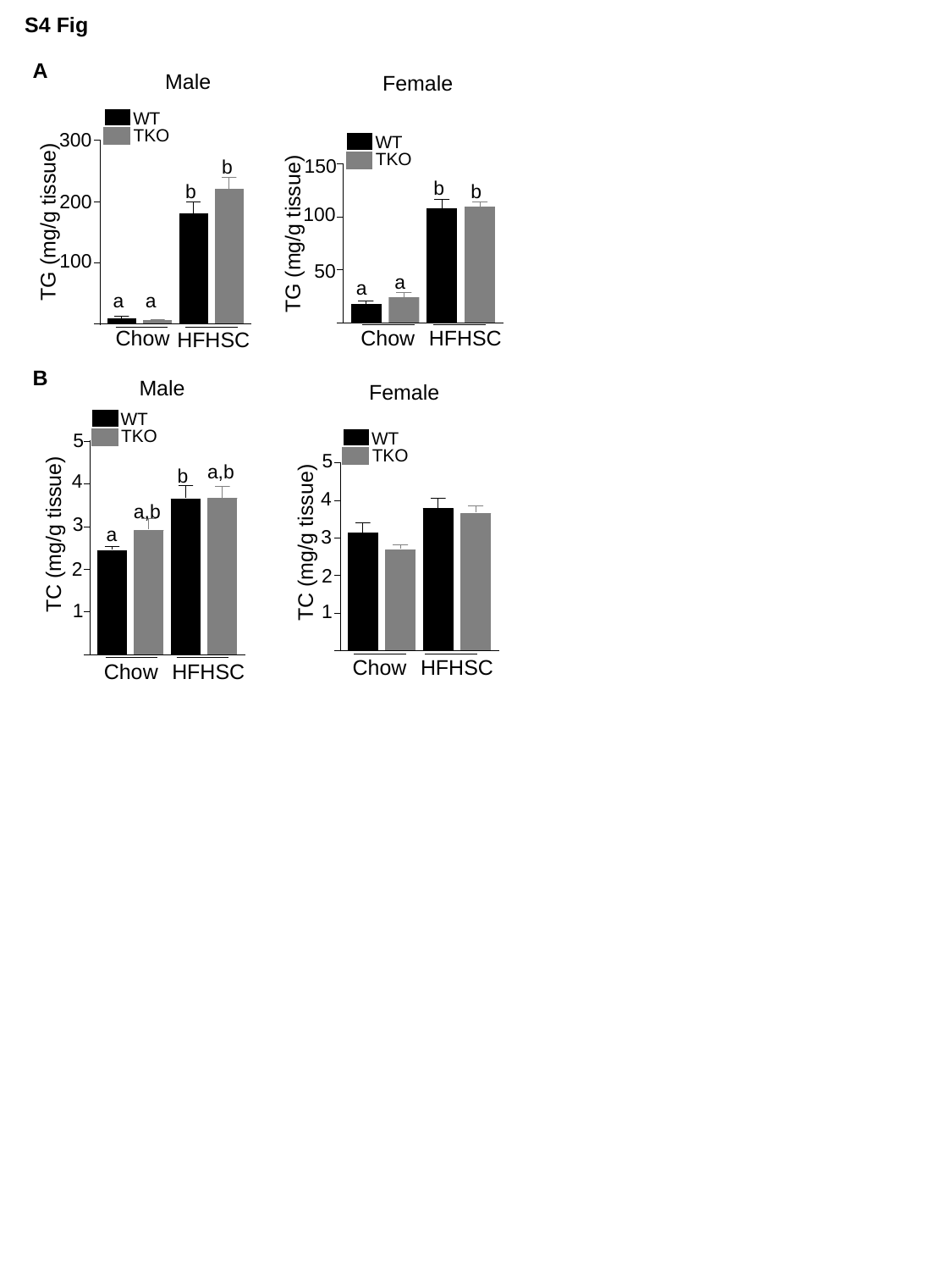

S4 Fig
A
Male
WT
TKO
300
b
b
200
TG (mg/g tissue)
100
a
a
Chow
HFHSC
Female
WT
TKO
150
b
b
100
TG (mg/g tissue)
50
a
a
Chow
HFHSC
B
Male
WT
TKO
5
a,b
b
4
a,b
3
TC (mg/g tissue)
a
2
1
Chow
HFHSC
Female
WT
TKO
5
4
3
TC (mg/g tissue)
2
1
Chow
HFHSC
