# Supplementary material for: Serum Amyloid A is not obligatory for high-fat, high-sucrose, cholesterol-fed diet-induced obesity and its metabolic and inflammatory complications
Source: PLoS One. 2022 Apr 18;17(4):e0266688. doi: 10.1371/journal.pone.0266688 (PMC9015120; doi:10.1371/journal.pone.0266688)
Supplement: S5 Fig — Expression of SREBP1-c, SCD-1, HMGCoAR and FAS genes (A-D) respectively in the livers of male (left panel) and female (right panel) WT and TKO mice fed either chow or HFHSC diet for 16 weeks (n = 4-10/group). Data are mean ±SEM; data that are not significantly different (P>0.05) are indicated with the same letter. (PPTX) [file pone.0266688.s005.pptx]

## Slide 1
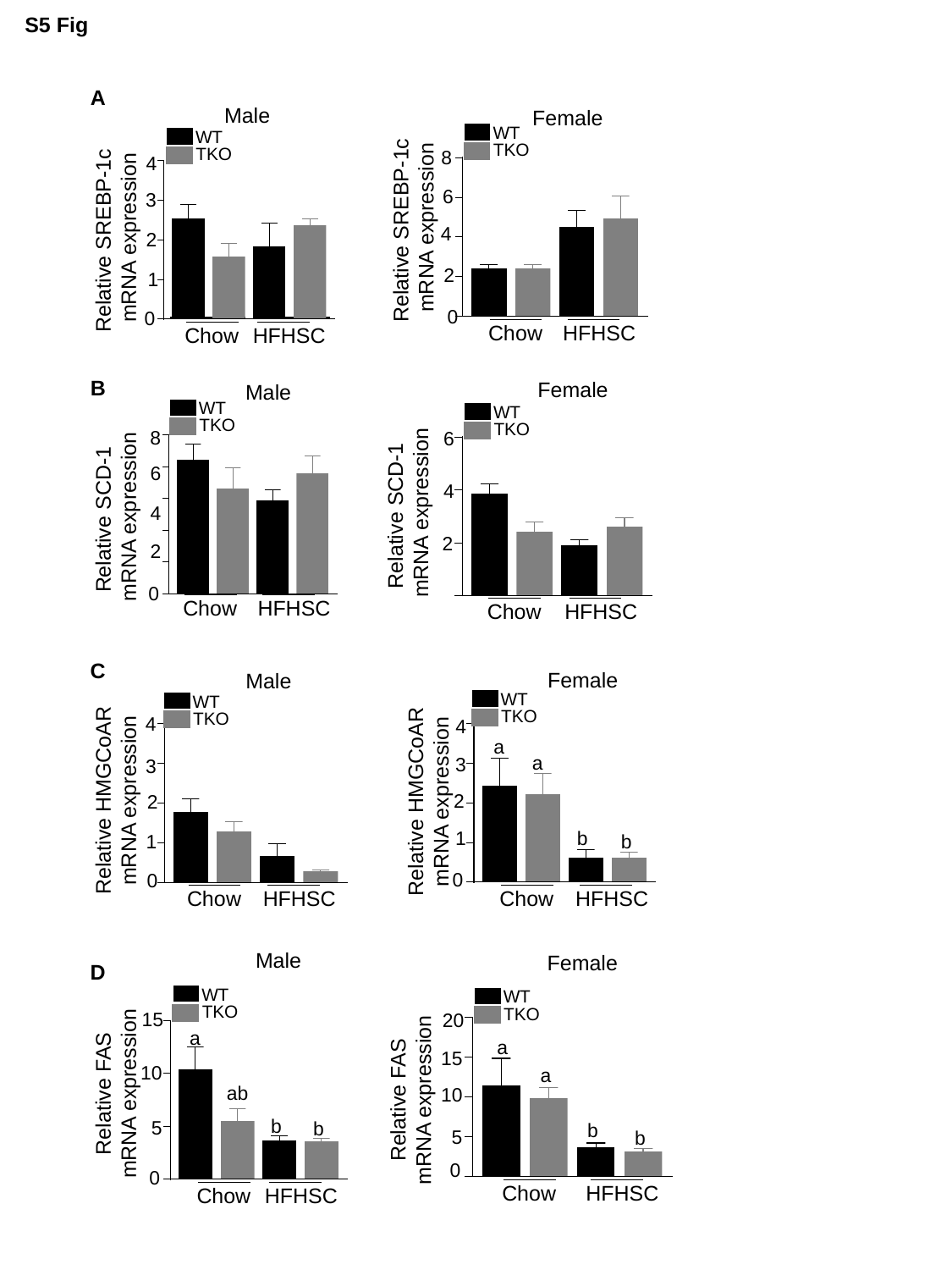

S5 Fig
A
Male
WT
TKO
4
3
Relative SREBP-1c
mRNA expression
2
1
0
Chow
HFHSC
Female
WT
TKO
8
6
Relative SREBP-1c
mRNA expression
4
2
0
Chow
HFHSC
B
Female
WT
TKO
6
4
Relative SCD-1
mRNA expression
2
Chow
HFHSC
Male
WT
TKO
8
6
Relative SCD-1
mRNA expression
4
2
0
Chow
HFHSC
C
Female
WT
TKO
4
a
a
3
Relative HMGCoAR
mRNA expression
2
1
b
b
0
Chow
HFHSC
Male
WT
TKO
4
3
Relative HMGCoAR
mRNA expression
2
1
0
Chow
HFHSC
Male
WT
TKO
15
a
10
Relative FAS
mRNA expression
ab
b
5
b
0
Chow
HFHSC
Female
WT
TKO
20
a
15
a
10
b
5
b
0
Chow
HFHSC
Relative FAS
mRNA expression
D
