# Supplementary material for: Serum Amyloid A is not obligatory for high-fat, high-sucrose, cholesterol-fed diet-induced obesity and its metabolic and inflammatory complications
Source: PLoS One. 2022 Apr 18;17(4):e0266688. doi: 10.1371/journal.pone.0266688 (PMC9015120; doi:10.1371/journal.pone.0266688)
Supplement: S6 Fig — Expression of IL-6 A) and IL-1β B) mRNA in the adipose tissues of male (left panel) and female (right panel) WT and TKO mice fed either chow or HFHSC diet for 16 weeks (n = 4-10/group). C) Plasma endotoxin levels at the study termination in male (left panel) and female (right panel) WT and TKO mice fed either chow or HFHSC diet for 16 weeks (n = 4-10/group). Data are mean ±SEM. (PPTX) [file pone.0266688.s006.pptx]

## Slide 1
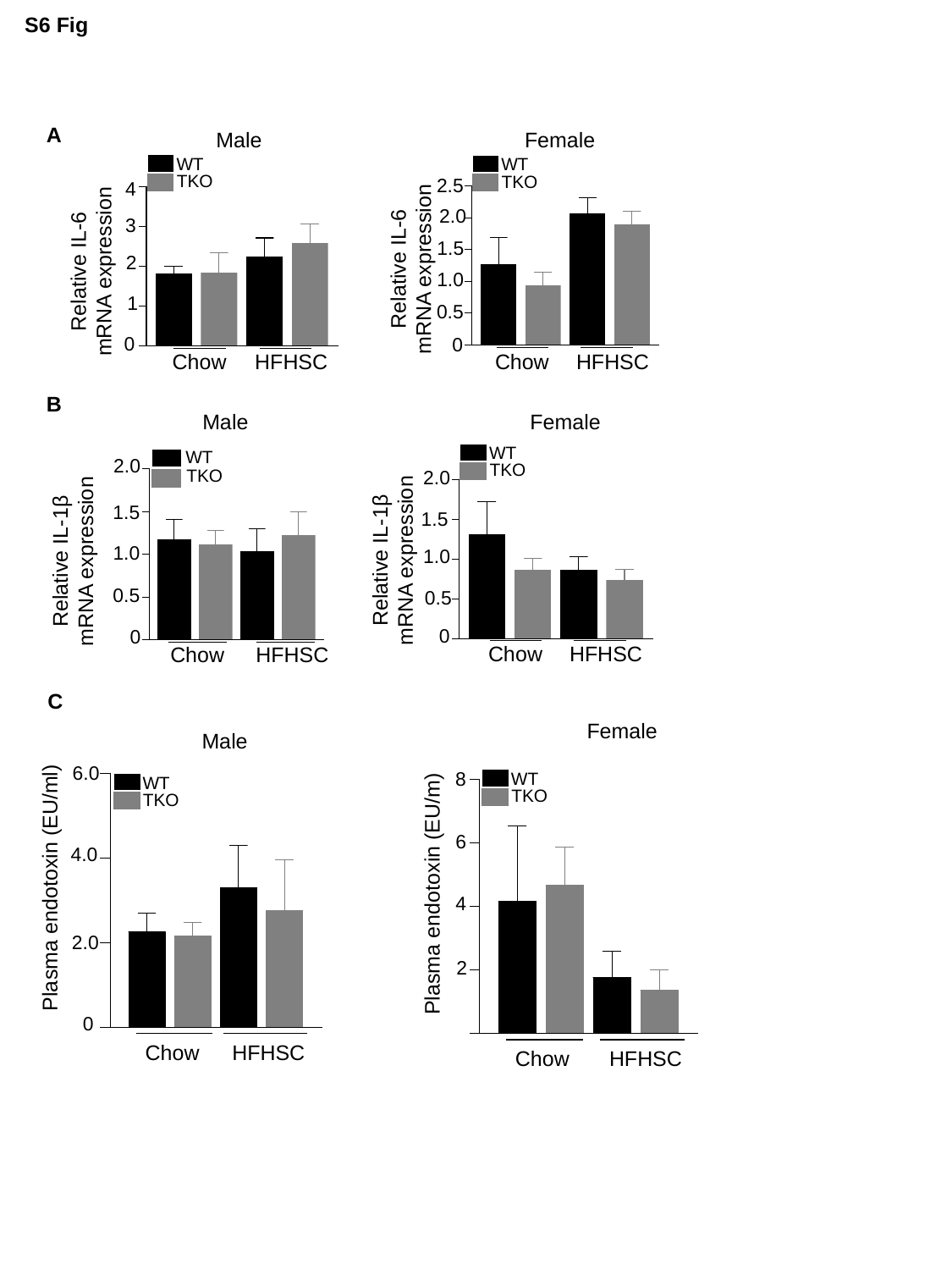

S6 Fig
A
Male
Female
WT
TKO
4
3
Relative IL-6
mRNA expression
2
1
0
Chow
HFHSC
WT
TKO
2.5
2.0
1.5
Relative IL-6
mRNA expression
1.0
0.5
0
Chow
HFHSC
B
Male
Female
WT
TKO
2.0
1.5
Relative IL-1β
mRNA expression
1.0
0.5
0
Chow
HFHSC
WT
TKO
2.0
1.5
Relative IL-1β
mRNA expression
1.0
0.5
0
Chow
HFHSC
C
Female
Male
8
Plasma endotoxin (EU/m)
4
2
Chow
HFHSC
WT
TKO
6
6.0
WT
TKO
4.0
Plasma endotoxin (EU/ml)
2.0
0
Chow
HFHSC
