# Supplementary material for: Serum Amyloid A is not obligatory for high-fat, high-sucrose, cholesterol-fed diet-induced obesity and its metabolic and inflammatory complications
Source: PLoS One. 2022 Apr 18;17(4):e0266688. doi: 10.1371/journal.pone.0266688 (PMC9015120; doi:10.1371/journal.pone.0266688)
Supplement: S1 Raw images — (PDF) [file pone.0266688.s007.pdf]

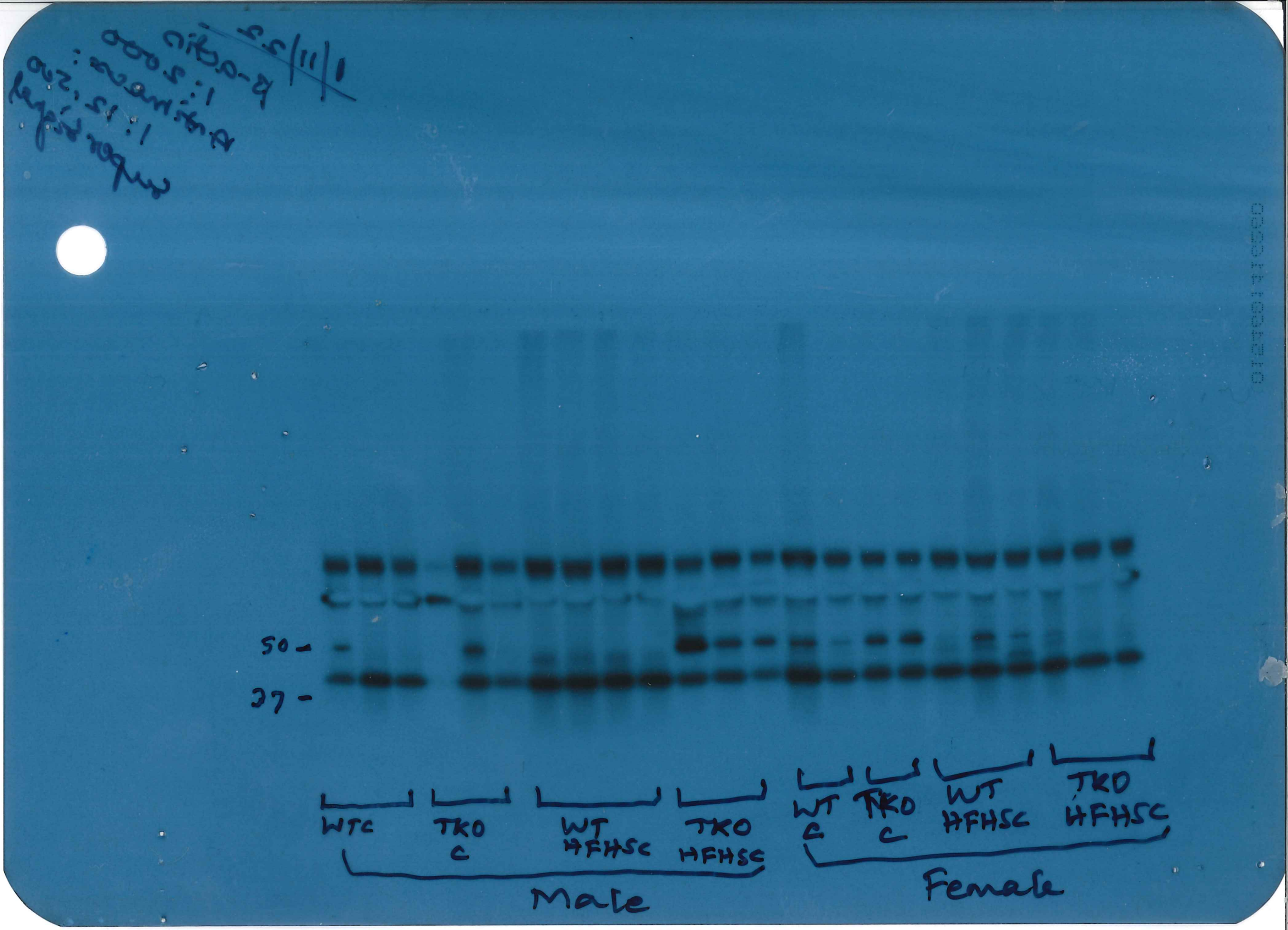

50
37
WTC
TKO C
WT HFHSC
TKO HFHSC
WT C
TKO C
WT HFHSC
TKO HFHSC
Male
Female

scWbg fat extract exp 022720 12/8/21

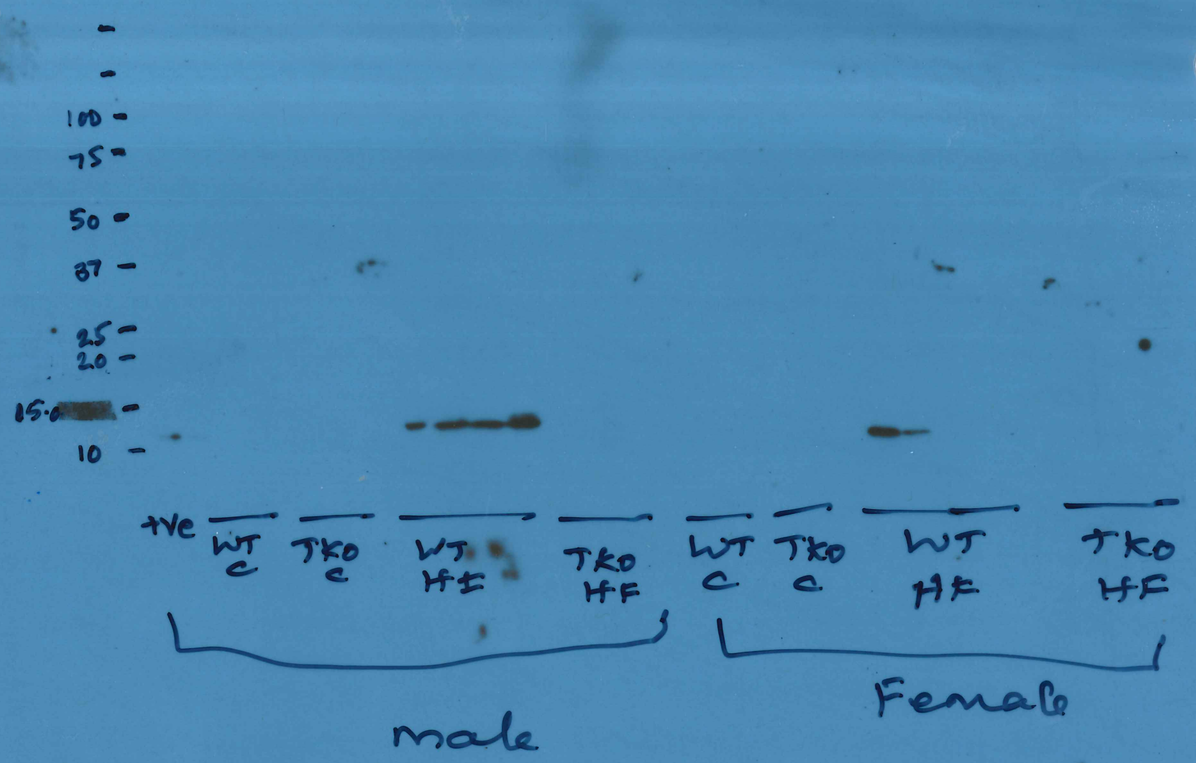

Advanced ECL - 10 min

1 : 1000 Abcam Anti SAA

1 : 25,000 anti Rb

EXP 022720

Subq Fat extract

Date: 12/7/21

Well	Sample	50µg [Protein] ~~(µg/µl)~~	Protein (µg) l	Buffer Volume (µl)	4 X SDS (µl)	Load (µl)
1	mol wt markers	—	—	—	—	15µl
2	+ve C (TgFSAA 1µl)		1	14	5µl	15µl
3	WT-C #1		8.9	6.1	↓	↓
4	" #2		12.5	2.5		
5	" #3		11.3	3.7		
6	TKO-C #7		11.0	4.0		
7	#8		8.1	6.9		
8	#9		11.7	3.3		
9	WT-HFHSC #12 (leaked)		10.1	4.9		
10	#12		10.1	4.9		
11	#14		11.1	3.9		
12	#15		10.9	4.1		
13	TKO-HFHSC #16		18.6	—		
14	#17		15.3	—		
15	#19		11.5	3.5		
16	WTC #26		10.4	4.6		
17	#27		9.3	5.7		
18	TKOC #32		8.4	6.6		
19	#33		8.3	6.7		
20	WT HFHSC #36		18.9	0		
21	37		15.9	0		
22	38		13.5	1.5		
23	TKO HFHSC # ~~18~~ 41		18.4	—		
24	#42		20.3	—		
25	43		20.2	—	↓	↓
26						

X 5-20% SDS ____4-18% GGE ____Other:

X Reduced ____Non-Reduced

X Boil Samples ____DO NOT BOIL

____Coomassie ____Transfer: 1° = Abcam SAA 1:1000 2° = 1:25,000 Antirabbit

____ ARG for ____

Gel label/orientation
