# Supplementary material for: Serum Amyloid A is not obligatory for high-fat, high-sucrose, cholesterol-fed diet-induced obesity and its metabolic and inflammatory complications
Source: PLoS One. 2022 Apr 18;17(4):e0266688. doi: 10.1371/journal.pone.0266688 (PMC9015120; doi:10.1371/journal.pone.0266688)
Supplement: S2 Raw images — (PDF) [file pone.0266688.s008.pdf]

12/01/21

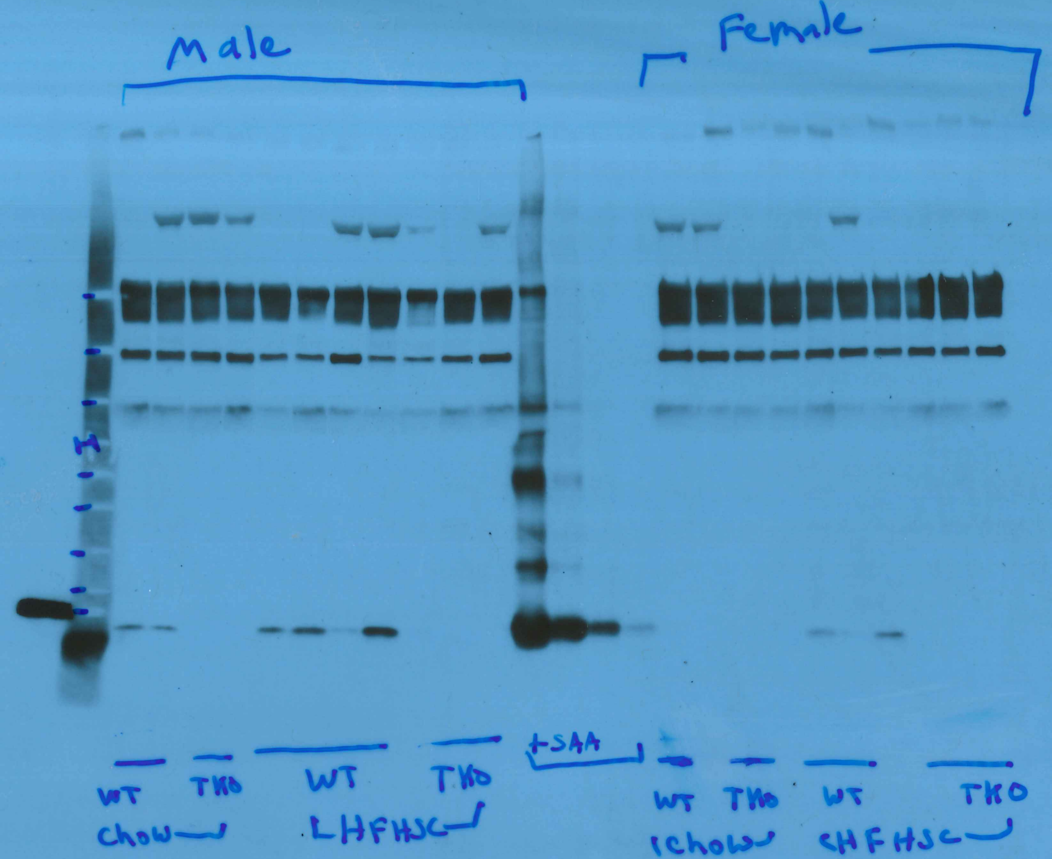

Run 200V 39min

Transfer 100V 38min

1° O/N 4°C

Rbα ms SAA3 sherer 1:1000

+ Rbα ms SAA1/2 abcam (199033) 1:1,000

-Wash 1L

2° α Rb HRP 1:10,000 1hr RT

~~Block~~ -Wash 1L

No Boil No Reduce

ECL 2min

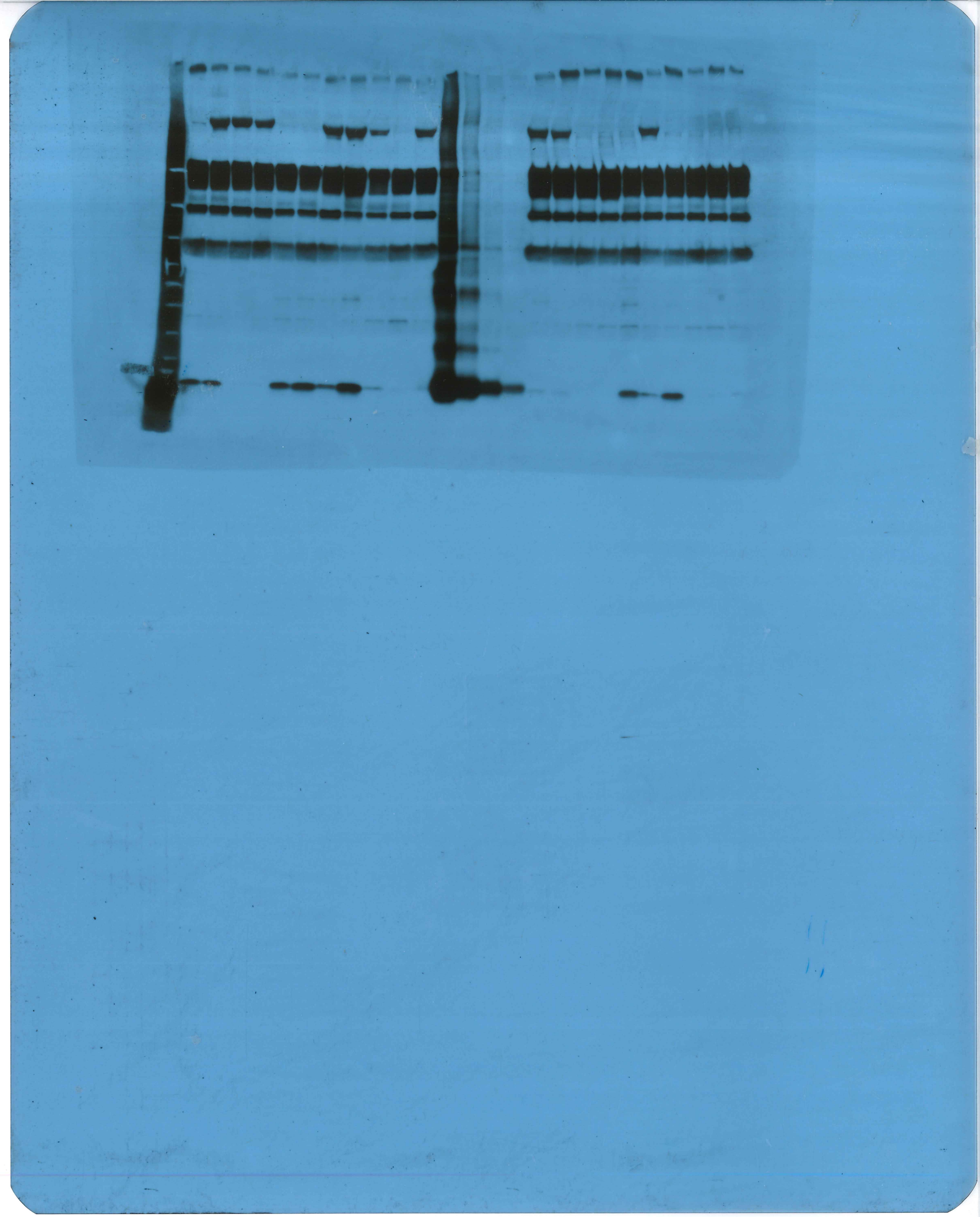

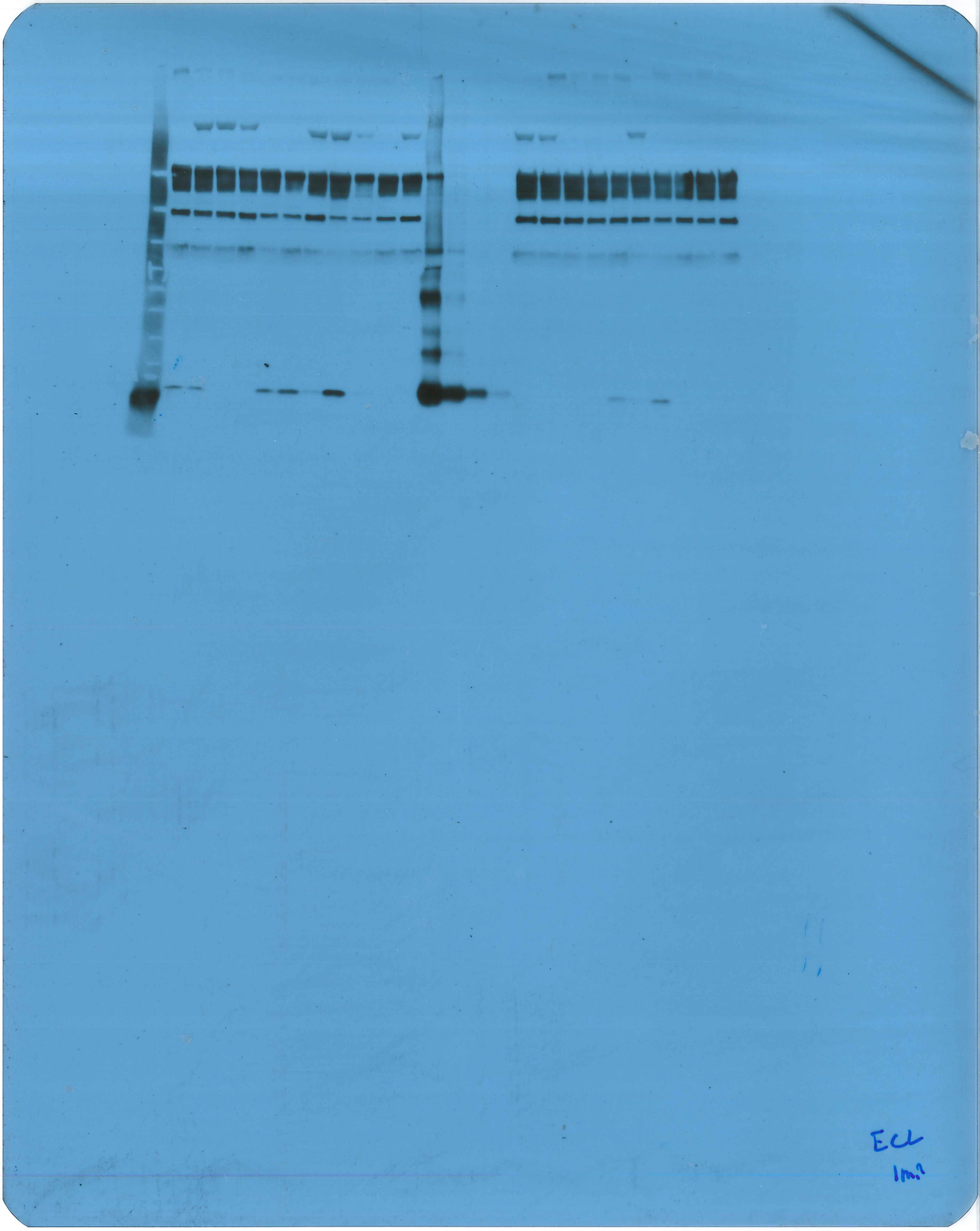
ECL
1mi

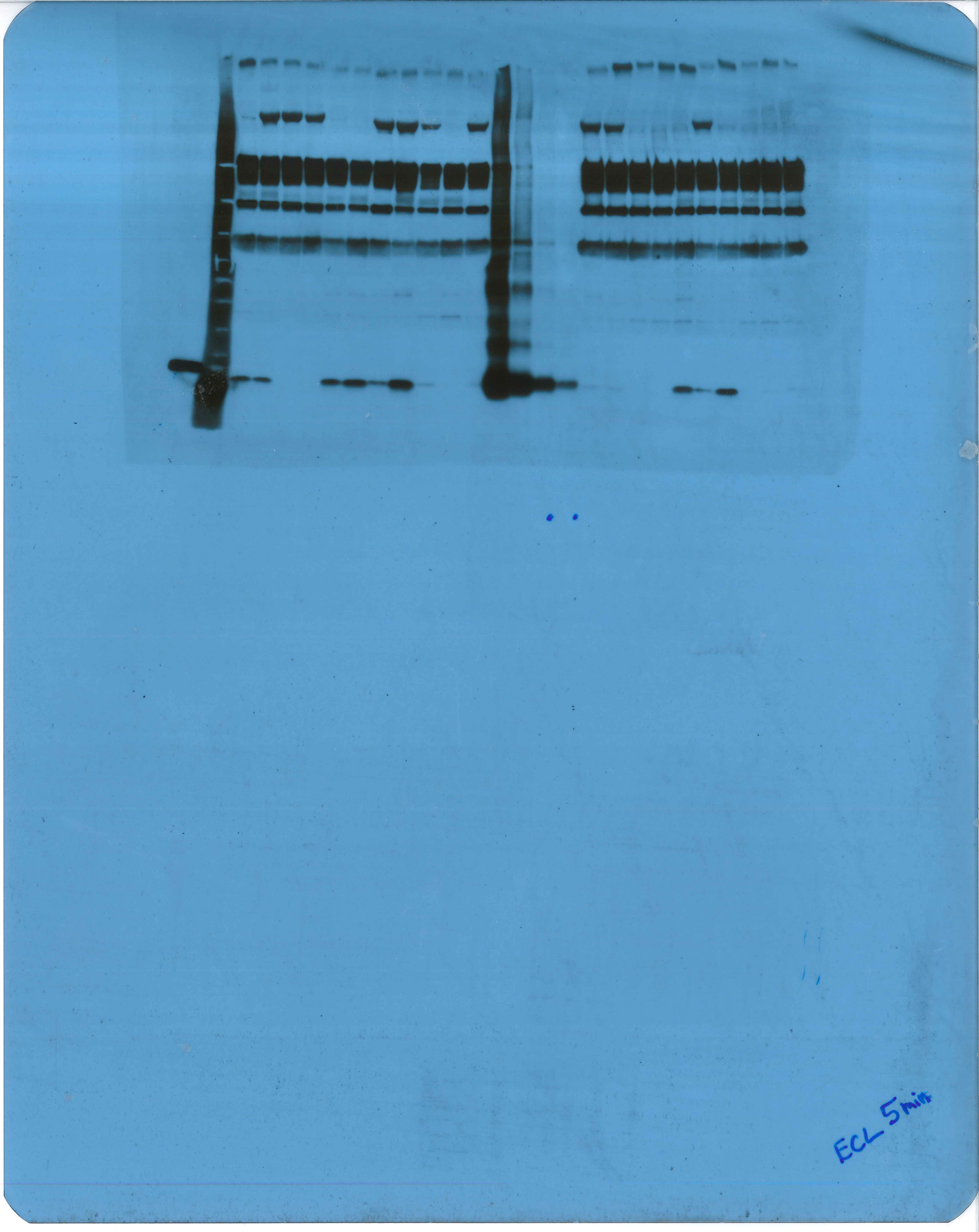
ECL 5min

Exp 022720 SAA 1/2/3 in plasma

Date: 11/30/21

Well	Sample	[Protein] (µg/µl)	Protein (µg)	Volume (µl)	2 X SDS (µl)	Load (µl)
1	Ladder					15
2	#3 WT chow M			3	27	10
3	#4 "					
4	#8 TKO chow					
5	#9					
6	#11 WT HF					
7	#12					
8	#14					
9	#15					
10	#16 TKO HF					
11	#18					
12	#19					
13	SAAAP A "			2	8	8 Bull 7
14	" B See ball			2	8	7
15	" C "			2	8	9
16	" D "			2	8	7
17	#27 WT chow F					8
18	#29 " "					
19	#32 TKO chow					
20	#33 " "					
21	#37 WT HF					
22	#38 WT HF					
23	#39					
24	#41 TKO HF					
25	#43					
26	#44					

____5-20% SDS ____4-18% GGE ____Other:

____Reduced ____Non-Reduced

____Boil Samples ____DO NOT BOIL

____Coomassie ____Transfer: 1° = Rb α ms SAA1/2 ab199030 1:1000 × Rb α SAA3 Scherer 1:1000 O/N 2° = α Rb HRP 1:10,000 1h

____ARG for ________

Gel label/orientation

APms plasma. ~~3+27ul~~

A Apms Plasma → [illegible] 2ul

B ↳ 10ul A + 30 2xSDS = 2ul

↳ 10ul B + 30 2xSDS = 2ul

↳ 10ul C + 30 2xSDS 2ul

↳

4/0
