# Supplementary material for: Serum Amyloid A is not obligatory for high-fat, high-sucrose, cholesterol-fed diet-induced obesity and its metabolic and inflammatory complications
Source: PLoS One. 2022 Apr 18;17(4):e0266688. doi: 10.1371/journal.pone.0266688 (PMC9015120; doi:10.1371/journal.pone.0266688)
Supplement: Attachment — Submitted filename: Response to Reviewers comment - PONE-D-21-32072.docx [file pone.0266688.s011.docx]

Response to Reviewer’s comment:

We thank the reviewers for their positive comments recognizing that the manuscript entitled “Serum Amyloid A is not obligatory for high-fat, high-sucrose, cholesterol-fed diet-induced obesity and its metabolic and inflammatory complications” (PONE-D-21-32072) has merit to be published in PLOS ONE. As requested, we provide a point-to-point response to all reviewer’s comments below. Changes in the resubmitted manuscript are indicated as yellow highlighted text (please see ’Revised manuscript with Track Changes’).

In the revised manuscript, we have added one more author, Avery C Patrick, who carried out some of the experiments suggested by the reviewers.

New and revised figures are described in our point-to-point response, below.

Response to Associate Editor’s comment:

1.Please review your reference list to ensure that it is complete and correct. If you have cited papers that have been retracted, please include the rationale for doing so in the manuscript text, or remove these references and replace them with relevant current references. Any changes to the reference list should be mentioned in the rebuttal letter that accompanies your revised manuscript. If you need to cite a retracted article, indicate the article’s retracted status in the References list and also include a citation and full reference for the retraction notice.

In response to the academic editor’s advice, we have reviewed the reference list and made sure that it is complete and correct. The list does not contain any retracted references.

Response to Reviewer’s comment

Reviewer 1:

Reviewer #1: The authors have provided responses and revisions to the manuscript that substantially improve the work. Nevertheless, the phenotype of the triple-SAA-knockout mice is still lacking robust endpoints. Specifically, the western blot raw data provided implicate that the only antibody used for the assessment of the SAA proteins (Figure 2B&D and supplemental material) was one from Abcam, which recognizes SAA1/2. Despite it being written in the results section, there is no indication that an SAA3-specific primary antibody was used. The rabbit anti-mouse SAA3 antibody from Dr. Scherer is going on 20 years old at this point, and it hasn't been available from him for a decade. It is not convincing that the results presented in the revised manuscript demonstrate an absence of SAA3 in these triple-SAA-knockout mice. The anti-SAA3 antibody should be used alone in a similar western blot. Alternatively, high-quality and specific SAA3 ELISAs are commercially available, and should be used to finally document the phenotype of these triple knockout mice as a complement to the SAA1/2 analysis already included.

We understand the concerns of the reviewer. In response to the concern, we have now included a new data showing the complete lack of SAA3 in the plasma of the triple-SAA-knockout mice (new Fig. 2F; results in page number 9, 2^nd^ paragraph, lines 9-11). The assay was performed with a SAA3-specific elisa kit, ZMSAA3-12K; EMD Millipore, Danvers, MA; the specificity of this elisa kit has been previously tested and reported; Tannock et al., J. Lipid Res. 2018. 59: 339–347. In addition, we have the data showing the absence of SAA3 mRNA in the adipose tissues as well as livers of TKO mice used in the experiment (S1B and S1D Figures respectively).

Reviewer #3: The revised manuscript is much improved from the earlier version and is responsive to the suggestions from the reviewers. In particular, the abstract now describes the main findings of the study and makes an appropriate conclusion.
Two minor points should be addressed:

(1) The authors have now measured adipocyte size in the wild type and triple knockout mice and claim that although there is a trend towards larger fat cell size in the knockout group (line 296), the differences were not statistically different. However, this conclusion is based on 3 views from only 2 mice in each group. Clearly, a greater samples size is required for adequate comparison between groups.

In response to the comment, we now have adipocyte size measurements from 3 views and 3 mice in each group. We conclude that there are no significant strain-dependent differences in adipocyte sizes with either diet and sexes (revised figure 7B). The method is revised in the ‘Materials and Methods’, under the sub-heading ‘Histology’, page number 7 and described in the ‘Results’ section, page number 13 (highlighted text).

(2) Line 275: In contrast to the study cited by Ather and Poynter that showed weight gain in Saa3 deficient mice, a study by den Hartigh et al showed that SAA3 deficient mice actually lost weight in response to an obesogenic diet (PMID: 25251243). This reference should also be included in this paragraph, even though they both are cited in the discussion section.

As suggested by the reviewer, we have now included the study results from de Hartigh et al in the results section of the revised manuscript, page number 12-13, lines 281-283.
